# Parental Views of Social Worker and Chaplain Involvement in Care and Decision Making for Critically Ill Children with Cancer

**DOI:** 10.3390/children9091287

**Published:** 2022-08-26

**Authors:** Kelly N. Michelson, Melanie Arenson, Elizabeth Charleston, Marla L. Clayman, Tracy Brazg, Karen Rychlik, Abby R. Rosenberg, Joel Frader

**Affiliations:** 1 Department of Pediatrics, Northwestern University Feinberg School of Medicine, Chicago, IL 60611, USA; 2 Center for Bioethics and Medical Humanities, Northwestern University Feinberg School of Medicine, Chicago, IL 60611, USA; 3 Division of Pediatric Critical Care Medicine, Ann & Robert H. Lurie Children’s Hospital of Chicago, Chicago, IL 60611, USA; 4 Department of Psychology, University of Maryland, College Park, MD 20742, USA; 5 Department of General Internal Medicine and Geriatrics, Northwestern University Feinberg School of Medicine, Chicago, IL 60611, USA; 6 Center for Healthcare Organization and Implementation Research (CHOIR), Veterans Health Administration, Bedford, MA 01730, USA; 7 Center for Health Sciences Interprofessional Education, Research and Practice, University of Washington, Seattle, WA 98195, USA; 8 Department of Bioethics and Humanities, University of Washington School of Medicine, Seattle, WA 98195, USA; 9 Stanley Manne Children’s Research Institute, Ann & Robert H. Lurie Children’s Hospital of Chicago, Chicago, IL 60611, USA; 10 Department of Pediatrics, Division of Hematology/Oncology, University of Washington School of Medicine, Seattle, WA 98195, USA; 11 Palliative Care and Resilience Lab, Seattle Children’s Research Institute, Seattle, WA 98145, USA; 12 Cambia Palliative Care Center of Excellence, UW Medicine, Seattle, WA 98104, USA; 13 Division of Pediatric Palliative Care, Ann & Robert H. Lurie Children’s Hospital of Chicago, Chicago, IL 60611, USA

**Keywords:** pediatric intensive care unit, social workers, chaplains, spiritual care providers

## Abstract

Background: Social workers (SWs) and chaplains are trained to support families facing challenges associated with critical illness and potential end-of-life issues. Little is known about how parents view SW/chaplain involvement in care for critically ill children with cancer. Methods: We studied parent perceptions of SW/chaplain involvement in care for pediatric intensive care unit (PICU) patients with cancer or who had a hematopoietic cell transplant. English- and Spanish-speaking parents completed surveys within 7 days of PICU admission and at discharge. Some parents participated in an optional interview. Results: Twenty-four parents of 18 patients completed both surveys, and six parents were interviewed. Of the survey respondents, 66.7% and 75% interacted with SWs or chaplains, respectively. Most parents described SW/chaplain interactions as helpful (81.3% and 72.2%, respectively), but few reported their help with decision making (18.8% and 12.4%, respectively). Parents described SW/chaplain roles related to emotional, spiritual, instrumental, and holistic support. Few parents expressed awareness about SW/chaplain interactions with other healthcare team members. Conclusions: Future work is needed to determine SWs’/chaplains’ contributions to and impact on parental decision making, improve parent awareness about SW/chaplain roles and engagement with the healthcare team, and understand why some PICU parents do not interact with SWs/chaplains.

## 1. Introduction

Cancer is the leading non-traumatic cause of death in children >1 year of age [1,2,3]. Forty percent of pediatric patients with cancer require treatment in the pediatric intensive care unit (PICU). Many of the children admitted to the PICU require invasive, life-sustaining therapies such as mechanical ventilation and vasoactive support [4,5]. Almost 30% of PICU patients with cancer will not survive—a 5-fold higher mortality rate than critically ill children without cancer [5,6,7,8,9].

Parents of children with cancer admitted to the PICU often face emotional and logistical hardships yet must remain sufficiently composed to make challenging decisions for their child and family. Parents may feel distress from witnessing their child struggle with critical illness and not knowing what the future holds for their child, themselves, and their family [10]. Some parents struggle to balance time spent with their critically ill child against the needs of other family members [11]. Within this context, parents are often asked to participate in decisions about their child’s care, such as whether to use or avoid a particular therapy. Parents must also make personal decisions about when and how to interact with the healthcare team, or whether to include other family members in their child’s or family’s care [12].

For these reasons, PICU care teams must address families’ social, behavioral, spiritual, informational, and environmental needs in addition to providing medical care for their critically ill child with cancer [11]. Social workers (SWs) and chaplains have extensive training in supporting people facing such adaptive challenges, i.e., challenges that are associated with non-technical aspects of patient/family relationships in the setting of medical complexity [13]. Experts have developed standards for the delivery of pediatric oncology psychosocial care, including recommendations for SW and chaplain support [14], yet these standards do not address the specific experiences and needs of PICU families [10,14]. Indeed, information about parents’ views on how PICU SWs and chaplains can support them during their child’s PICU admission is limited. In this hypothesis-generating, descriptive, multi-methods study, we analyzed parents’ views of their interactions with SWs and chaplains during their child’s PICU admission and how parents perceive SW and chaplain involvement in decision making for their critically ill child with cancer.

## 2. Methods

### 2.1. Study Environment

This study took place between July 2014 and April 2017 at an academic children’s hospital 40-bed PICU staffed by PICU attendings, fellows, pediatric residents, advanced practice nurses, bedside nurses, and others (e.g., respiratory therapists, child-life specialists). As part of usual care, 1 of 5 SWs and 1 chaplain followed patients with cancer and patients post hematopoietic cell transplant (HCT) on weekdays starting at the time of diagnosis. The SW and the chaplain followed patients and families throughout their illness regardless of the patients’ location (outpatient, on the medical floor, or in the PICU). Evenings and weekends were covered by on-call SWs and chaplains. In addition to SW- and chaplain-initiated visits, parents could request SW or chaplain involvement. All participants gave their informed consent prior to the study. The hospital institutional review board approved the study, which was conducted in accordance with the principles embodied in the Declaration of Helsinki.

### 2.2. Study Participants

Parents of children in the PICU were eligible to enroll in the study if they spoke English or Spanish, if their child had either a cancer diagnosis or had undergone an HCT, and if the child had been in the PICU < 7 days. We included patients after HCT (even those with nonmalignant hematologic indications) because they experience similar processes of care and medical and psychosocial situations prior to and during PICU admission as patients with cancer (e.g., consequences of pre-transplant conditioning, post-transplant immunosuppression, and multi-organ dysfunction). To identify a patient population with relatively high morbidity and mortality, we included parents of patients with 1 or more of the following: Pediatric Index of Mortality 2 (PIM2) score ≥ 4%, expected PICU admission of >3 days (based on attending PICU physician input), previous PICU admission, plan to consult palliative care, or involvement of palliative care. We used convenience sampling and identified and enrolled parents during weekdays. Multiple parents of the same patient could participate in the study. Data collection was halted intermittently secondary to a conflicting study with similar enrollment criteria.

### 2.3. Data Collection

Enrolled parents completed 2 surveys (using certified Spanish translations as needed). The enrollment survey asked about parent demographics, religiosity, spirituality, and social support. Religiosity/spirituality was assessed using 2 previously studied “yes/no” questions: “Is religion an important part of your daily life?” and ‘‘Do you consider yourself a spiritual person?’’ [15,16]. Social support was assessed using the Information Support-Short Form 8a (Support SF 8a) PROMIS measure, an 8-item survey that utilizes a 5-point Likert scale to measure the perceived availability of helpful information or advice provided by others (alpha reliability of 0.95) [17]. Responses are scored (1 = never, 2 = rarely, 3 = sometimes, 4 = usually, 5 = always), summed, and then converted to a t-score (a standardized score with a mean of 50 representing the average United States population score) [17].

The second survey, given within 3 days of PICU discharge, asked parents if they spoke with an SW and/or chaplain during the PICU admission, and if so, whether the SW and/or chaplain helped them. Parents rated their experience with the SW and/or chaplain as “excellent,” “very good,” “good,” “fair,” or “poor,” and were asked if they would speak with an SW and/or chaplain again if a future PICU admission occurred. Items were tested for clarity and face validity by an SW, chaplain, PICU clinician, and 2 experts in communication and decision making (K.N.M. and M.L.C.). The second survey also asked parents to (1) identify “the most important decision made for their child in the PICU,” (2) complete the Decision Regret Scale (DRS), a measure of regret about the identified decision [18,19], (3) indicate who on the healthcare team helped them talk with their doctors about that decision, and (4) complete 10 items from the Pediatric Family Satisfaction in the Intensive Care Unit (pFS-ICU) Survey related to decision making [20]. The DRS includes 5 items scored on a 5-point Likert scale (1 = strongly agree, 2 = agree, 3 = neither agree nor disagree, 4 = disagree, 5 = strongly disagree). Scoring involves reversing the scores of 2 negatively phrased items, taking the mean, then converting that mean to a 0–100 score (0 indicates no regret and 100 the highest regret) [18]. The DRS shows good internal consistency (Cronbach’s alpha 0.81 to 0.92) and correlates strongly with decision satisfaction (r = −0.4 to −0.6), decisional conflict (r = 0.31 to 0.52), and quality of life (r = −0.25 to −0.27) [19]. The pFS-ICU decision-making domain (hereafter referred to as satisfaction with decision making [SDM]) has a Cronbach’s alpha of 0.88 and includes 10 items scored on a Likert scale from 1–5 (1 = poor, 2 = fair, 3 = good, 4 = very good, 5 = excellent). Scores are averaged and transformed into a 0–100 score (0 indicates the least satisfaction and 100 the most satisfaction) [21].

Parents were also invited to participate in an optional 1-on-1 semi-structured interview at PICU discharge. Interviewed parents were asked if they had interacted with SWs or chaplains in the PICU and whether and how those interactions were helpful. Parents were also asked about their views regarding how SWs and chaplains interacted with the rest of the healthcare team and participated in making decisions the parents encountered (see Parent interview guide, Table S1). All interviews were conducted in English (no Spanish-speaking parents volunteered to be interviewed), audio-recorded, and transcribed. In 2 cases, the audio recorder malfunctioned, and notes were used for analysis.

We extracted the following information from the medical record: patient demographics, PIM2, PICU length of stay (LOS), type of cancer, indication for PICU admission, introduction of palliative care, use of continuous renal replacement therapy (CRRT) or extracorporeal membrane oxygenation (ECMO), new “do not attempt resuscitation” (DNAR) order or other orders limiting life-sustaining therapies, and death in the PICU.

### 2.4. Data Analysis

We analyzed quantitative data using descriptive statistics, expressing continuous variables as mean, median, and standard deviation (SD) and categorical variables as counts and percentages. This was a hypothesis-generating study and was not powered to make statistical comparisons or test hypotheses. Two authors (K.N.M. and E.C.) reviewed all decisions identified by parents and created categories of decisions. The same 2 authors labeled each decision by category separately, compared labels, and resolved differences by consensus.

We analyzed interview data using directed content analysis of verbatim deidentified transcripts or notes [22]. Two people (M.A. and E.C.) separately reviewed the transcripts and created code lists with associated definitions. The code lists were narrowed through discussion, eliminating redundancy, and combining similar codes. The revised list was used to code the remaining transcripts, creating new codes as necessary. We identified subsets of the data related to 3 topics: (1) SW/chaplain interactions with parents, (2) SW/chaplain interactions with the healthcare team, and (3) SW/chaplain involvement in decision making. We used SAS version 9.4 (SAS Institute Inc., Cary, NC, USA) for quantitative analysis and Dedoose (Dedoose Version 5.2.0, 2014, SocioCultural Research Consultants, LLC, Los Angeles, CA, USA) for qualitative analysis.

## 3. Results

We screened 1382 patients; the parents of 92 patients were eligible for inclusion in the study. We invited the parents of 41 patients (who were present during weekdays when a study team member was available) to participate. Thirty-six parents of 28 patients (68%, 28/41) agreed to participate. Of the participating parents, 24 parents of 18 patients (44%, 24/41) completed both surveys and were included in this analysis (Table S2 provides demographics and clinical characteristics of eligible, invited, and enrolled patients). Six parents of 6 patients also completed an interview. Table 1 and Table 2 show demographic information about parent participants and their critically ill children, respectively, for all parents and those who reported interactions with SWs and chaplains. Three parents (12.5%, 3/24) (of 3 patients) reported no interaction with either an SW or chaplain. No study patients required CRRT or ECMO, had a new DNAR order or a new order describing limitations of life-sustaining therapies, or died.

### 3.1. Survey Responses

#### 3.1.1. Experiences with SW and Chaplains

Table 3 shows parent responses to questions about SW and chaplain interactions. Sixteen (66.7%) and 18 (75%) parents reported speaking with an SW or chaplain, respectively. Of those 16 parents, 13 (81.3%) described the SW as helpful, 14 (87.5%) rated their experience as excellent, and 13 (81.3%) would ask to speak with an SW again if in the PICU. Of the 18 parents who spoke with a chaplain, 13 (72.2%) described the chaplain as helpful, 9 (50.0%) rated their experience as excellent, and 15 (83.3%) would ask to speak with a chaplain again if in the PICU.

#### 3.1.2. Parent Decision-Making Support

Parent-reported decisions included decisions related to the use of a medical device (8, 13.3%), the child’s general care plan (4, 16.7%), use of particular medications (3, 12.5%), a surgical procedure (2, 8.3%), and use of radiation (1, 4.2%) (Table 3). Sixteen (66.7%) parents reported that someone helped them talk with doctors about the decision identified. Most parents identified the bedside nurse as helping discuss decisions (10, 62.5%). Only 3 (18.8%) and 2 (12.5%) parents identified the SW or chaplain, respectively, as helping them discuss decisions.

#### 3.1.3. Parent Decision Regret and Decision-Making Satisfaction

Table 4 shows the DRS scores, stratified by parent-reported involvement with an SW or chaplain. The mean DRS score for all decisions was 9.8 (median 0, SD 15). The mean DRS was highest for “not specified” decisions (mean 20, median, 10, SD 26.5), decisions related to “surgical procedures” (mean 17.5, median 17.5, SD 24.7), “use of medications” (mean 15, median 5, SD 21.8), and “general care plan” (mean 15, median 17.5, SD 10.8). The mean DRS for parents who reported involvement with SWs was 11.7 (median 0, SD 17.4) compared to 6.7 (median 5, SD 7.5) for those who did not report SW involvement. The mean DRS for parents who reported involvement with chaplains was 10.9 (median 0, SD 16.4) compared to 2.5 (median 0, SD 5) for those who did not report chaplain involvement.

The mean SDM score for all parents was 82.2 (median 90, SD 16.1). The mean SDM for parents who reported involvement with SWs was 84.8 (median 90, SD 16.1) compared to 72.1 (median 68.8, SD 18.5) those who did not report SW involvement. The mean SDM for parents who reported involvement with chaplains was 82.4 (median 90, SD 17.5) compared to 81.9 (median 80, SD 14) those who did not report chaplain involvement.

### 3.2. Parent Interviews

Parent interviews provided depth and context to the survey data. All 6 parents who were interviewed described interactions with an SW, and 4 described interactions with a chaplain. In the following sections, the numbers in parenthesis represent parent or patient participant identification numbers.

#### 3.2.1. SW and Chaplain Emotional and Spiritual Support

The majority of parent comments described both SWs and chaplains as providing important emotional support for themselves or their child during a very difficult time. About an SW, 1 parent said,

*“No, I mean she was just, she was a calming presence. You know she would just come in once and a while and check in on us, see if we needed anything.”*

(005)

That same parent reflected on how conversations with the SW were often not related to medical issues and that was comforting.

*“I mean it was just nice to have somebody who didn’t come and talk to us about you know medical things. You know just someone who would be like “how are you doing?”*

(005)

The 4 parents who interacted with a chaplain also described receiving emotional support. One parent said,

*“The chaplain was involved while (015) was in the PICU, and would stop to chat about what was going on medically with (015), how I was feeling. Made sure I wasn’t alone…”*

(015)

One parent noted that the chaplain helped them feel more hopeful about their situation.

*“You know it’s like you never give up and that’s one thing that the chaplain said.”*

(014)

Parents also described how the chaplains provided spiritual support. Some parents noted that the chaplains prayed with them and for their child. One parent said,

*“She (the chaplain) was very specific in her prayers when she prayed for us. We said like our traditional prayers but then she made it a little more personable, too, and she used my daughter’s name and she said, keep the medical team’s hands steady when they put—you know, so she made it personal instead of just a random prayer…”*

(002)

#### 3.2.2. SW and Chaplain Instrumental and Holistic Support

Along with emotional support, parents described receiving instrumental, i.e., more tangible, support, as well as holistic support that tended to their broader psycho-social-spiritual needs.

Five parents noted how SWs helped provide resources or logistical support, such as finding a sleeping room, helping with parking or food vouchers, finding financial support, managing work issues (e.g., using the Family Medical Leave Act), and even supporting siblings or peers. One parent described the myriad ways the SW helped navigate resources and made space to talk about her other children,

*“… she helped me with parking, food vouchers, Ronald McDonald housing. We talked about a lot of things as far as even with my, about my other daughter. She helped me for her and with my other son. She helped also with because I’m not working, I’ve been out of work since my son got sick so she also helped me with a grant that would help me out with my bills.”*

(019)

One parent noted,

*“She (the SW) is helping with resources to educate [my child’s] peers at school this month.”*

(006)

Discussing a chaplain, 1 parent said,

*“And, then they [the chaplain] came over and they gave her like a little prayer and she carried that prayer everywhere and she had like a little rosary.”*

(014)

#### 3.2.3. SW and Chaplain Involvement in Medical Decision Making

Three of the parents noted that interactions with SWs or chaplains did not impact their decision making. One parent felt their interaction with the SW was a barrier to decision making. That parent described how the SW gave them unwanted information about their child.

*“You know it’s like I didn’t feel like they were actually helping me, … you know it’s like you just felt so overwhelmed. You know and then for them you know just to be coming in here and giving us information of what if this happens, you know what if you, you know your daughter doesn’t make it. It’s like that’s the last thing that you want to hear.”*

(014)

#### 3.2.4. SW and Chaplains as Part of the Healthcare Team

When asked about SW and chaplain interactions with other healthcare team members, most (4 of 6) parents were uncertain if such interactions occurred. Several described that SWs and chaplains exist separately from the rest of the team. One parent said,

*“I guess I didn’t see a whole lot of interaction between the medical and the SWs. … I guess I was never thinking … that [the SW] could help me with something that had to do with my interactions with the nurses or someone in the PICU versus just kind of helping us with more like resources.”*

(005)

One parent noted that they did not feel there was a reason for SWs or chaplains to interact with the rest of the healthcare team. Four parents felt that it would be good for the SWs and chaplains to share aspects of their interactions with the healthcare team.

*“Yes, I think I would want them to. You know it’s like what I think they should be sharing is like, especially the way my daughter was acting, … I’m not sure what the, or how the chaplains did it. For some reason my daughter felt comfortable with them. … but I think that would be nice if they were to talk among each other and maybe it was something they said to her you know that made her feel very comfortable with them. ….”*

(014)

One parent explicitly described how an SW helped her address communication concerns with the medical team. That parent said,

*“But I had these concerns. I brought them to the social worker. The social worker brought them to the managers. They all sat with me, they all talked with me…”*

(002)

## 4. Discussion

In this multi-methods study, most, but not all, parents reported speaking with an SW or chaplain during their PICU stay and described those experiences as positive. Few parents reported that SWs or chaplains helped them talk with doctors about important decisions. Mean DRS scores were higher (i.e., more decision regret) in parents who interacted with SWs or chaplains. Mean satisfaction with decision making scores were higher (i.e., more satisfaction with decision making) for parents who interacted with an SW. We cannot comment on whether these differences were statistically significant. Interviewed parents described SW and chaplain roles related to emotional, spiritual, information, and holistic support, only a few noted SW and chaplain involvement in decision making, and there was minimal understanding about SW and chaplain interactions with other healthcare team members.

Parents’ perceptions of SW and chaplain roles mirrored some of those reported in the literature. In adult-care settings, SWs provide concrete resources, act as patient counselors and advocates, help patients and families cope with stress, and may improve physician-family communication [23,24,25,26]. In pediatrics, SWs provide psychosocial support, act as counselors, and guide families through decisions [27,28]. In some settings, chaplains act as ethicists; therapists; and counselors to patients, families, and the medical team; in addition to participating in care discussions [29,30,31,32]. Parent participants in this study noted that SWs and chaplains focused more on providing emotional, spiritual, instrumental, and holistic support, and were less involved in decision making and care discussions.

Given the literature describing the role of SWs and chaplains in care discussions, we did not expect that parents who interacted with SWs and chaplains would, on average, report more decision regret. One explanation for this result may be that SWs and chaplains interacted more with families as they were facing difficult decisions, and such decisions are associated with higher decision regret. We also found that, on average, parents who interacted with SWs reported higher satisfaction with the decision-making process. Perhaps the support provided by SWs and chaplains related less to communicating about decisions and more about tending to the emotional, spiritual, or even logistical issues parents faced while making difficult decisions. We acknowledge that any trends seen in this study must be viewed as hypothesis-generating, that decisional regret among parents of children with cancer is complicated [33], and that interactions with SWs and/or chaplains may not be the driving factors that affect decision regret or satisfaction. Our results do suggest that additional research is needed to better understand the impact of SW and/or chaplain involvement on decision regret and satisfaction.

This work highlights important improvement opportunities. Guidelines recommend providing interprofessional psychosocial support for children with cancer throughout the care trajectory [14,34]. Yet, 12.5% of parents did not interact with an SW or chaplain during their child’s PICU stay, arguably a key time to provide families with psychosocial support. We hypothesize that one explanation for why all parents did not report these interactions may be that some parents felt they did not need additional help or did not know how SWs or chaplains could help. Two parents reported not knowing what SWs do, and 4 parents did not know if SWs or chaplains interacted with the rest of the team. Another possibility is that SWs and chaplains have limited availability. We know that the case load for SWs can be as high as 60 or more patients per month [24]. Future work should explore what types of support parents feel best meets their needs, including how SWs and chaplains should provide such support.

Based on our results and existing literature, we recommend the following to improve engagement with SWs and chaplains. First, educate parents about the roles of SWs and chaplains and how they interact with the medical team. The importance of interprofessional involvement is known, but less is known about how to inform parents about healthcare team collaboration [14]. Second, educate healthcare providers about SW and chaplain roles. Role ambiguity and blurred boundaries are commonly cited by hospital SWs as barriers to interprofessional collaboration [35,36]. Research also demonstrates limited physician knowledge about the breadth of services and care provided by chaplains [37]. Education of healthcare providers might help mitigate these issues and enhance SW and chaplain referrals. Third, make offering support from an SW and/or chaplain to all PICU parents a quality metric. We realize that not everyone may want or benefit from SW and/or chaplain involvement while in the PICU. Notably, 1 parent in this study reported a negative experience with an SW. Thus, monitoring both positive and negative outcomes of such interactions and whether both SWs and chaplains are needed are additional important quality metrics. Finally, while this study focused on the roles of SWs and chaplains in the PICU, it is important to remember that SWs and chaplains also have a critical role in the care of patients with cancer from the point of their diagnosis and during all phases of treatment, not just in the PICU.

We acknowledge some study limitations. Participants were largely female, white, and married parents. The study was conducted at a single site between 2014–2017 where patients with cancer were followed throughout their care trajectory by the same SW and chaplain, it is possible that practice may have changed since the study was conducted. Not all hospitals use this staffing model. We have a relatively small sample size in part because we had to pause enrollment due to a conflicting study. As a relatively small study, we cannot comment on data saturation (i.e., we may not have described the full range of SW and chaplain roles), and we could not report comparative statistics. Our data are subject to response bias; notably, the parents of only 44% of eligible patients who were invited enrolled in the study. These issues limit generalizability. Finally, we only presented parent perspectives. Future work should include input from other members of the PICU healthcare team, including SWs and chaplains.

In conclusion, most parents of PICU patients with cancer in our study interacted with an SW and/or chaplain and viewed those experiences positively. However, more research is needed to understand why some PICU parents do not interact with SWs or chaplains and the possible contributions of SWs and chaplains in the care and decision-making process for parents. Attention to the integration of and parent awareness about SW and chaplain engagement with the larger healthcare team is also needed.

## Figures and Tables

**Table 1:** Parent demographic characteristics.

	All Parents (*N* = 24)	Parents Reporting Interactions with Social Workers (*N* = 16)	Parents Reporting Interactions with Chaplains (*N* = 18)
Gender, *N* (%)			
Female	17 (70.8)	12 (75.0)	14 (77.8)
Male	7 (29.2)	4 (25.0)	4 (22.2)
Race, *N* (%)			
Asian	1 (4.2)	1 (6.2)	0 (0)
Black	1 (4.2)	1 (6.2)	1 (5.6)
White	17 (70.8)	10 (62.5)	13 (72.2)
Other	5 (20.8)	4 (25.0)	4 (22.2)
Ethnicity, *N* (%)			
Not Hispanic/Latinx	13 (54.2)	6 (37.5)	10 (55.5)
Hispanic/Latinx	11 (45.8)	10 (62.5)	8 (44.4)
Education, *N* (%)			
Elementary school	2 (8.3)	1 (6.2)	1 (5.6)
High school	8 (33.3)	6 (37.5)	7 (38.9)
College	7 (29.2)	6 (37.5)	5 (27.8)
Post-graduate	5 (20.8)	3 (18.8)	3 (16.6)
Other education	2 (8.3)	0 (0)	2 (11.1)
Marital Status, *N* (%)			
Living as a married couple	3 (12.5)	2 (12.5)	3 (16.7)
Married	15 (62.5)	3 (18.8)	10 (55.5)
Separated	1 (4.2)	1 (6.2)	1 (5.6)
Single	5 (20.8)	5 (31.2)	4 (22.2)
“Religion Is an Important Part of My Daily Life” ^A^, *N* (%)	19 (79.2)	11 (68.8)	14 (77.8)
“I Consider Myself a Spiritual Person,” *N* (%)	23 (95.8)	15 (93.8)	17 (94.4)
Age ^B^, mean (median, SD)	36 (36.1, 8.1)	35.3 (35.7, 7.6)	35 (35.7, 8.0)
Information social support, mean (median, SD)	55.9 (55.4, 8.8)	54.9 (54.2, 8.3)	55.6 (56.1, 8.9)

SD, standard deviation. ^A^, missing response from one parent; ^B^, missing response from three parents.

**Table 2:** Patient demographic and clinical characteristics.

	All Patients (*N* = 18)	Patients Whose Parents Interacted with Social Workers (*N* = 13)	Patients Whose Parents Interacted with Chaplains (*N* = 15)
Sex, *N* (%)			
Female	6 (33.3)	3 (23.1)	4 (26.7)
Male	12 (66.7)	10 (76.9)	11 (73.3)
Age, mean (median, SD)	7.4 (7.2, 4.5)	7.6 (7.5, 4.6)	6.6 (7.5, 5.0)
PIM2, mean (median, SD)	2.5 (2.7, 1.8)	2.0 (1.1, 1.4)	2.16 (1.1, 1.6)
PICU LOS, mean (median, SD)	8.1 (6, 6.6)	8.9 (6.5, 7.4)	8.8 (6, 6.9)
Cancer or post HCT, *N* (%)			
Hematologic cancer	7 (38.8)	3 (23.1)	4 (26.7)
HCT	4 (22.2)	3 (23.1)	4 (26.7)
Solid tumor	7 (38.8)	7 (53.8)	7 (46.6)
Indication for PICU admission, *N* (%)			
Hemodynamic instability	5 (27.8)	3 (23.1)	3 (20.0)
Neurologic dysfunction	4 (22.2)	3 (23.1)	3 (20.0)
Postoperative care	5 (27.8)	4 (30.7)	4 (26.7)
Respiratory failure	4 (22.2)	3 (23.1)	4 (26.7)
Palliative care initiated during PICU admission, *N* (%)			
Yes	3 (16.7)	3 (23.1)	3 (20.0)
No	15 (83.3)	10 (76.9)	12 (80.0)

HCT, hematopoietic cell transplant; LOS, length of stay; PICU, pediatric intensive care unit; PIM2, Pediatric Index of Mortality 2; SD, standard deviation.

**Table 3:** Parent survey responses.

Question	Response	*N*	%
Did you speak with a social worker? (*N* = 24)	Yes	16	66.7
	No	6	25.0
	Missing	2	8.3
Did the social worker help you in any way? (*N* = 16)	Yes	13	81.3
	No	0	0
	Missing	3	18.8
How would you rate your experience with the social worker overall? (*N* = 16)	Excellent	14	87.5
	Very Good	0	0
	Good	1	6.3
	Fair	1	6.3
	Poor	0	0
	Missing	0	0
Would you ask to speak with a social worker again? (*N* = 16)	Yes	13	81.3
	No	0	0
	Missing	3	18.8
Did you speak with a chaplain? (*N* = 24)	Yes	18	75.0
	No	4	16.7
	Missing	2	8.3
Did the Chaplain help you in any way? (*N* = 18)	Yes	13	72.2
	No	5	27.8
	Missing	0	0
How would you rate your experience with the chaplain overall? (*N* = 18)	Excellent	9	50.0
	Very Good	1	5.6
	Good	6	33.3
	Fair	1	5.6
	Poor	0	0
	Missing	1	5.6
Would you ask to speak with a chaplain again? (*N* = 18)	Yes	15	83.3
	No	1	5.6
	Missing	2	11.1
What was the most important decision made for your child in the PICU? (*N* = 24)	Use of medical device	8	13.3
	General care plan	4	16.7
	Use of medication	3	12.5
	Surgical procedure	2	8.3
	Use of radiation	1	4.2
	Not specified	4	16.7
	None	2	8.3
Was there anyone on the healthcare team who helped you talk with your doctors about the decision made for your child? (*N* = 24)	Yes	16	66.7
	No	5	20.8
	Missing	3	12.5
Who was involved? (*N* = 16) ^A^	Bedside nurse	10	62.5
	Doctor/APN ^B^	4	25.0
	Social worker	3	18.8
	Chaplain	2	12.5

APN, advanced practice nurse; PICU, pediatric intensive care unit. ^A^, some parents identified more than one person; ^B^, includes attendings, residents, fellows, and APNs.

**Table 4:** Parent Decision Regret Scale (DRS) scores.

	All Parents		Social Worker Involved		Social Worker Not Involved		Chaplain Involved		Chaplain Not Involved	
Type of Decision	N	Mean (Median, SD)	N	Mean (Median, SD)	N	Mean (Median, SD)	N	Mean (Median, SD)	N	Mean (Median, SD)
All responses	22	9.8 (0, 15.0)	15	11.7 (0, 17.4)	6	6.7 (5.0, 7.5)	17	10.9 (0, 16.4)	4	2.5 (0, 5.0)
Use of medical device	8	1.9 (0, 3.7)	4	0 (0, 0)	3	5 (5.0, 5.0)	6	2.5 (0, 4.2)	2	0 (0, 0)
General care plan	4	15 (17.5, 10.8)	2	20 (20.0, 7.1)	2	10 (10.0, 14.1)	2	20 (20.0, 7.1)	1	0 (0, NA)
Not specified	3	20 (10.0, 26.5)	3	20 (10.0, 26.5)	0	NA	2	25 (25.0, 35.4)	1	10 (10.0, NA)
Use of medication	3	15 (5.0, 21.8)	2	20 (20.0, 28.3)	1	5.0 (5.0, NA)	3	15 (5.0, 21.8)	0	NA
Surgical procedure	2	17.5 (17.5, 24.7)	2	17.5 (17.5, 24.7)	0	NA	2	17.5 (17.5, 24.7)	0	NA
Use of radiation	1	0 (0, NA)	1	0 (0, NA)	0	NA	1	0 (0, NA)	0	NA
None	1	0 (0, NA)	1	0 (0, NA)	0	NA	1	0 (0, NA)	0	NA

SD, standard deviation; NA, not applicable.

## Data Availability

The data are not publicly available due to participant confidentiality.

## Supplementary Materials

The following supporting information can be downloaded at: https://www.mdpi.com/article/10.3390/children9091287/s1, Table S1: Parent interview guide; Table S2: Demographics and clinical characteristics of eligible, invited, and enrolled patients.
