# Supplementary material for: Parental Views of Social Worker and Chaplain Involvement in Care and Decision Making for Critically Ill Children with Cancer
Source: Children (Basel). 2022 Aug 26;9(9):1287. doi: 10.3390/children9091287 (PMC9497868; doi:10.3390/children9091287)
Supplement: Supplementary file 1 [file children-09-01287-s001.zip › children-1862398-supplementary.pdf]

Table S1. Parent interview guide

Topic	Sample Question[A]
Understand SWs'/Cs' interactions with parents	Tell me about your interactions with the SW/C while your child was in the PICU?
	How did the SW/C help you or your child while your child was in the PICU?
Understand SWs'/Cs' interactions with the healthcare team	Did the SW/C help you interact with the medical team while your child was in the PICU?
	Should SWs/Cs share information regarding their interactions with you or your child with the medical team? And if so what and how?
Understand decisions and the decision-making process for parents	What was the most important decision made for your child while s/he was in the PICU?
	What involvement did you have in the decisions made for your child while he/she was in the PICU?
	How did your interactions with the SW/Chaplain affect the decision-making process for you?" (Applicable only for those who interacted with a SW or C)

C, chaplain; PICU, pediatric intensive care unit; SW, social worker. [A]The exact questions could change depending on the flow of the interview.

Table S2. Demographics and clinical characteristics of eligible, invited, and enrolled patients

	All Eligible Patients (N=92)	All Patients Whose Parent(s) Were Invited to Participate (N=41)	All Patients Whose Parent(s) Returned Both Surveys (N=18)
Sex, N (%)			
Female	39 (42.4)	18 (43.9)	6 (33.3)
Male	53 (57.6)	23 (56.1)	12 (66.7)
Age, mean (median, SD)	8.7 (8.7, 5.2)	7.5 (8.0, 5.5)	7.4 (7.0, 4.5)
PIM2, mean (median, SD)	3.3 (3.2, 3.4)	3.0 (2.5, 3.3)	2.5 (2.7, 1.8)
Cancer or status post HCT, N (%)			
Hematologic cancer	44 (47.8)	17 (41.4)	7 (38.9)
SCT	14 (15.2)	7 (17.2)	4 (22.2)
Solid tumor	34 (37.0)	17 (41.4)	7 (38.9)
Indication for PICU Admission, N (%)			
Hemodynamic instability	29 (31.5)	13 (31.7)	5 (27.8)
Neurologic dysfunction	23 (25.0)	9 (22.0)	4 (22.2)
Postoperative care	17 (18.5)	8 (19.5)	5 (27.8)
Respiratory failure	23 (25.0)	11 (26.8)	4 (22.2)

HCT, hematopoietic cell transplant; PICU, pediatric intensive care unit; PIM2, Pediatric Index of Mortality 2; SD, standard deviation.
